# Water Content Monitoring in Water-in-Crude-Oil Emulsions Using an Ultrasonic Multiple-Backscattering Sensor

**DOI:** 10.3390/s21155088

**Published:** 2021-07-27

**Authors:** Alberto L. Durán, Ediguer E. Franco, Carlos A. B. Reyna, Nicolás Pérez, Marcos S. G. Tsuzuki, Flávio Buiochi

**Affiliations:** 1 Laboratory of Computational Geometry, Escola Politécnica da Universidade de São Paulo, São Paulo 05508-220, Brazil; carlosburbano@usp.br (C.A.B.R.); mtsuzuki@usp.br (M.S.G.T.); fbuiochi@usp.br (F.B.); 2 Facultad de Ingeniería, Universidad Autónoma de Occidente, Santiago de Cali 760030, Colombia; eefranco@uao.edu.co; 3 Facultad de Ingeniería, Universidad de la Republica, Montevideo 11200, Uruguay; nico@fisica.edu.uy

**Keywords:** water-in-crude-oil emulsion, water content, ultrasound, propagation speed

## Abstract

This work shows the application of an ultrasonic multiple-scattering sensor for monitoring water-in-petroleum emulsions. The sensor consists of a commercial ultrasonic transducer with an array of cylindrical scatterers placed in the near field. The scatterers are thin metal bars arranged in rows in front of the transducer. The backscattering signals were analyzed by calculating the wave energy and by a cross-correlation between signal segments; they were also used to determine the propagation velocity in the emulsions. The tests performed used emulsions with water volume concentrations from 0% to 50%. The results showed that both the signal energy and propagation velocity strongly depended on the concentration of water in the emulsion. Therefore, the ultrasonic multiple-scattering sensor can be used for on-line and real-time monitoring of the water content in water-in-crude-oil emulsions.

## 1. Introduction

An emulsion is a mixture of two immiscible liquids, where one liquid is dispersed in the other in the form of small drops. Water-in-oil (W/O) and oil-in-water (O/W) emulsions are present in different industrial products and processes. Examples of final products in emulsion form include homogenized milk [1] and the cutting fluids used for machining processes [2]. There are also some processes in which the raw or intermediate product is an emulsion, such as palm oil [3] and petroleum production [4].

Some physical properties of the emulsion are relevant for controlling and improving the industrial process. The main property is likely the volume fraction of the constituent liquids (phases), because other properties, such as the emulsion type (W/O or O/W), stability, density, absorptivity, viscosity, etc., strongly depend on the volume fraction.

In the petroleum industry, crude oil extracted from a field has mixed water in different proportions. In the cases of offshore fields or enhanced oil recovery (EOR), in which water is used as displacement fluid to extract the remaining oil in old fields, the amount of water is generally greater. Its presence produces catalyst poisoning in the refining process, and if it is salty, additional corrosion problems are generated in pipes and equipment. In addition, the limit on the amount of water allowed in crude oil for transportation and refining is less than 1% [5].

Characterization techniques for oil-water emulsions include electron microscopy [6], light [7] and neutron [8] scattering, and nuclear magnetic resonance [9]. However, these techniques do not work well for dilute and opaque emulsions, such as water-in-crude-oil emulsions. Other techniques that perform better in opaque emulsions use X-rays [10] and gamma rays [11]. Yet ionizing radiation requires special handling. The best established techniques are likely the electrical methods, which are based on the measurement of the electrical conductivity, dielectric permittivity, electrical capacitance, and admittance [12,13].

These techniques are affected by the salinity and conductive properties of the mixture. In turn, common methods for determining the water content in crude oil, such as centrifugation [14], Karl Fisher distillation, and grinding methods [15], require extracting a sample from the pipelines for further processing in a laboratory. These laboratory tests are time-consuming and delay the processing or transportation of the crude oil.

Ultrasonic methods have also been used to determine the volume fraction of the constituent phases of emulsions, which are based on the ultrasonic measurement of the reflection, transmission and attenuation coefficients. These are interesting techniques due to the following advantages: the medium can be opaque; there is no ionizing radiation; they have relatively low costs; and the sensors and equipment are robust [16]. However, the propagation of acoustic waves in emulsions is a complex phenomenon that depends on the physical properties, the volume fraction of the phases, and the drop size distribution of the dispersed phase [17].

In a non-scattering medium, the attenuation is a consequence of the sound absorption through mechanisms that convert wave energy into heat, such as viscous losses, heat conduction losses, and relaxation losses [18]. In a scattering medium, an excess of attenuation and changes in the propagation velocity depend on the droplet size, the volume fraction, and the operating frequency. However, according to the scattering theory, the change in the propagation velocity due to scattering is small at the limit of the long wavelength. In turn, a change in concentration affects the propagation velocity to a greater extent [19,20]. This provides a useful measurement range for concentration monitoring using ultrasonic waves.

The literature has reported several works related to the characterization of water–oil mixtures by ultrasound. Ultrasound has been proven useful to detect water contamination in lubricating oils [21] and, conversely, to detect small amounts of oily substance in water [22]. To determine the concentration of the phases in the mixture, several approaches have been investigated. Ultrasonic spectroscopy allows determining the distribution of droplet sizes and concentration by analyzing the propagation velocity and attenuation spectra [17,23]. This is a well-established technique that is useful in a wide range of droplet sizes but restricted to dilute emulsions [24].

Water-in-crude-oil emulsions are concentrated with a wide drop size distribution [25]. This characteristic leads to dispersive and high attenuation media. Under these conditions, the best approach is the monitoring of an average acoustic parameter at a suitable operating frequency. In this sense, some authors have used the monitoring of the received amplitude [26] and the propagation velocity [27] as a measurement mechanism, using a variety of techniques for analyzing the signals. Those researchers studied the response of ultrasonic sensors (in transmission-reception mode) in a vertical channel.

Using symbolic dynamic filtering for the analysis of the signals, they showed the potential of the proposed technique to determine the water content in a water–oil mixture for water concentrations below 35% by volume [26]. Other authors reported experiments with mixtures of water and crude oil in a flow loop. A sensor based on acoustic methods allowed measuring the water concentration (between 64% and 96% of water by weight) with an error of less than 2%, compared to the concentration calculated with the mass flow of each phase measured by Coriollis-type sensors [28].

Ultrasonic backscattering has been used to measure the sediment concentration in liquids [29] and directly in rivers [30]. High frequency (50 MHz) ultrasonic backscattered signals were used for measuring cell concentration in cell cultures, showing that this technique allowed for the differentiation of red blood cells of various sizes [31]. The technique was also used for measuring the concentration of yeasts in suspensions, showing better sensitivity than spectrophotometric techniques [32]. A multiple-backscattering measurement sensor in the configuration used herein was used to monitor milk coagulation [33] and to characterize water-in-hydraulic-oil emulsions [34].

We report the application of a multiple-scattering ultrasonic sensor for monitoring water-in-crude-oil emulsions. The main interest is the online determination of the water content. Measurement with emulsion samples with water contents ranging from 0 to 50% in volume were carried out at $22{\;}^{\circ }$C. The cross-correlation and wave energy parameters were analyzed as a function of time and as a function of the water concentration. Additionally, a novel signal processing methodology for measuring the propagation velocity is proposed.

## 2. Materials and Methods

### 2.1. Measurement Device

**Figure 1:**
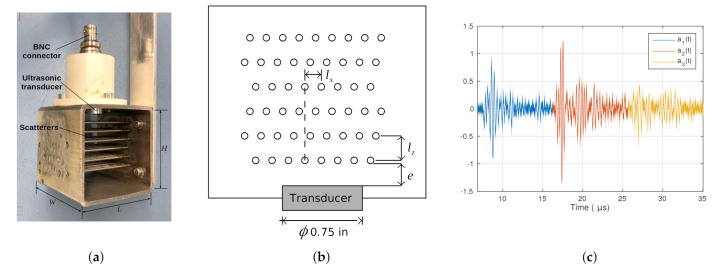
Multiple-scattering sensor: (**a**) image, (**b**) schematic representation of the distribution of the cylindrical scatterers in front of the transducer, and (**c**) acquired backscattering signal in water.

Figure 1a shows the measurement device, which consisted of a 3.5-MHz and 0.75-inch-diameter commercial ultrasonic transducer (Panametrics V381-SU, Olympus NDT INC, Waltham, MA, USA ) with an array of cylindrical scatterers placed in the near field. The configuration of the scatterers is shown in Figure 1b. The scatterers were made of stainless steel bars of 1.6-mm diameter, which were arranged in rows in a square stainless steel tube, where W=H=60 mm and L=65 mm. The separation between the rows was $l_{z}=6.0$ mm, and the separation between scatterers of the same row was $l_{x}=4$ mm. These rows were displaced one third of the separation between scatterers of a same row (4/3 mm), and thus the fourth row was aligned with the first. A polymeric adapter held the ultrasonic transducer.

Figure 1c shows the a(t) scattering signal obtained with the sensor. The $a_{1}\left(t\right)$, $a_{2}\left(t\right)$, and $a_{3}\left(t\right)$ signals are segments of a(t), defined around the theoretical arrival time of reflections from the first, second, and third row of scatterers, respectively. These theoretical arrival times ($t_{in}$) were defined using the design separation between consecutive rows of scatterers ($l_{z}$) and assuming the propagation velocity of water ($c_{w}$) at the test temperature ($22{\;}^{\circ }$C):

(1) $$t_{in}=\frac{2nl_{z}}{c_{w}},$$

where n=1,2,3 is the *n* th row of scatterers.

### 2.2. Signal Processing

In this work, we analyzed the effect of the water content in the emulsion on the backscattering ultrasonic signals. Several methodologies were used to extract the information from the signals. These methodologies are based on monitoring the change of a signal property over time, in relation to the signal obtained with a reference sample. The reference sample used in all tests was pure crude oil.

#### 2.2.1. Cross-Correlation

The cross-correlation measures the similarity of two signals as a function of the displacement of one relatively to the other [35]. It is defined by

(2) $$R\left[a_{ref},a\right]=\int_{-\infty }^{+\infty }a_{ref}\left(\tau \right)a\left(\tau +t\right)d\tau ,$$

where $a_{ref}\left(t\right)$ is the reference signal and a(t) the signal obtained with the emulsion. The result is normalized using the autocorrelation:

(3) $$R_{r}=\frac{R[a_{ref},a]}{R[a_{ref},a_{ref}]},$$

then, $R_{r}$ is denoted as the “normalized relative cross-correlation”.

#### 2.2.2. Relative Wave Energy

The wave energy is defined as the integral of the squared voltage signal and is calculated by

(4) $$E=\int_{t_{a}}^{t_{b}}{\left|a\left(t\right)\right|}^{2}dt,$$

where $t_{a}$ and $t_{b}$ define the calculation time window, covering the reflections from all rows of scatterers. Then, the energy of the signal obtained with the emulsion is normalized with the energy of the reference signal, as follows

(5) $$E_{r}=\frac{E}{E_{ref}}$$

#### 2.2.3. Propagation Velocity

To determine the propagation velocity, a signal analysis methodology that looks for the position of two consecutive rows of scatterers was implemented. A peak search algorithm seeks the arrival time of the maximum absolute value of amplitude. This is done in a time window centered around the theoretical arrival time (water case) of the reflections from the first ($a_{1}$) and second ($a_{2}$) rows of scatterers. The time difference ($\Delta t_{12}$) enables the propagation velocity to be calculated:

(6) $$c_{12}=\frac{2l_{z}}{\Delta t_{12}}.$$

The $l_{z}$ value is known. Small errors in the manufacturing process can modify the design value, and the calculated propagation velocity can be affected. To reduce this error, the $l_{z}$ distance was determined by performing a test with water at the temperature of interest, since the water propagation velocity is a well-known property.

The time delay calculation between the reflections from the first and second rows of scatterers can also be performed using an algorithm based on the cross-correlation. However, the results from this method showed a high dispersion. The peak search algorithm also showed deviations caused by the detection of a neighboring peak point. In this case, the results that could lead to wrong interpretations can be easily filtered because the obtained speed is typically far from the expected value. Another option is to take the value that appears most often (the mode).

### 2.3. Experimental Procedure

The samples were water-in-crude-oil emulsions with the volume concentration of water from 0% to 50%. The emulsions were made using a light crude oil supplied by the Brazilian company Petrobras ($30.5^{\circ }$ API), degassed water, and a mixer (Ika Labortechnik, Staufen, Germany, 8000–24,000 rpm) for homogenization. The samples, for each concentration, were mixed for 2 min at 8000 rpm, and then left to rest for 3 to 5 min to eliminate the air bubbles introduced in the mixing process. In the experiment, successive concentrations were obtained by adding water, at $22{\;}^{\circ }$C, to an emulsion that was previously prepared. We started with pure oil (water concentration of 0) and ended by achieving a water concentration of 50%.

**Figure 2:**
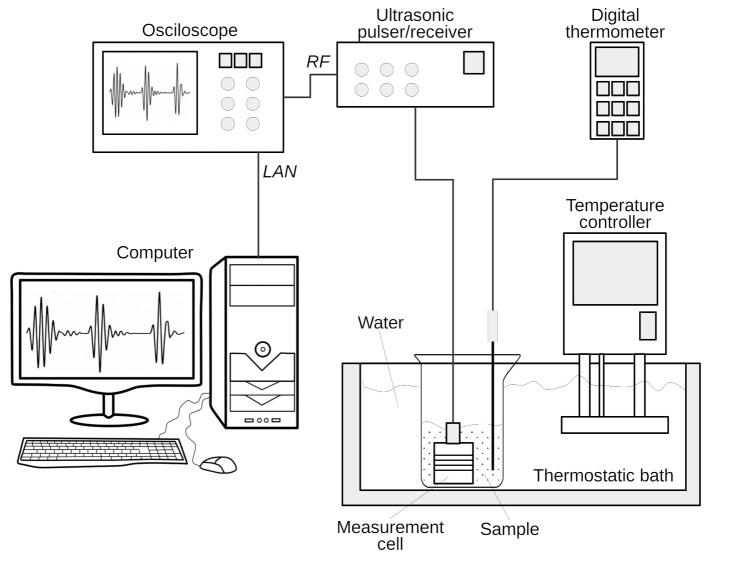
The measurement setup scheme.

Figure 2 shows the scheme of the measurement setup. An ultrasonic pulser–receiver (Panametrics 5072PR, Olympus NDT INC, Waltham, MA, USA) was used to drive a 3.5-MHz ultrasonic transducer (Panametrics V381, Olympus NDT INC, Waltham, MA, USA), operating in pulse-echo mode. The pulser–receiver unit was connected to an oscilloscope (DSO 5052A, Keysight Technology, Santa Rosa, CA, USA) to visualize and acquire the signals. The data was transferred to a desktop PC, via a LAN, for storage and further processing.

The measurement process was carried out in immersion, using a thermostatic bath (CC-104A, Huber Kältemaschinenbau AG, Offenburg, Germany) with temperature control and accuracy of $0.05{\;}^{\circ }$C. A glass beaker was immersed in the bath, containing the emulsion and the sensor. Additionally, the emulsion temperature was constantly monitored using a digital thermometer. After a stabilization time of about 15 min, the sample temperature reached the thermostatic bath setpoint and stayed in the range of $22.0\pm 0.2{\;}^{\circ }$C for the remaining test time.

Regardless of the measurement technique used, temperature and fluid movement are parameters that must be taken into account when characterizing water-in-crude-oil emulsions. The purpose of this work is to determine if the proposed ultrasonic device is sensitive to changes in the concentration of the emulsion and to propose measurement methodologies. For this reason, the work began under controlled conditions (room temperature and static emulsion).

Two types of tests were performed: long (2 h to almost 14 h) and short (15 min) term tests. The acquisition rate was approximately 1.67 signals per minute, where each recorded signal was the mean of 256 raw signals.

## 3. Results and Discussion

**Figure 3:**
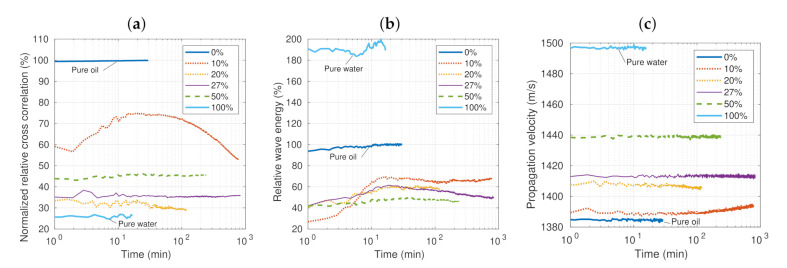
Experimental results of the long-term test at $22\pm 0.2{\;}^{\circ }$C: the relative cross-correlation (**a**), relative wave energy (**b**), and propagation velocity (**c**) as a function of time.

Figure 3 shows the measured acoustic parameters for the long-term test. Six concentrations were analyzed: pure water, pure crude oil, and four emulsions with a water concentration between 10% and 50% at $22\pm 0.2{\;}^{\circ }$C. The measurement times were dissimilar, from 16 min for the pure oil to 13 h for some emulsions. The normalized relative cross-correlation ($R_{r}$) and the relative wave energy ($E_{r}$) as a function of time are plotted in Figure 3a,b, respectively. The reference substance was pure oil. This test was carried out to analyze the stability of the measurements. The results show an almost linear behavior in the case of pure oil and water and nonlinear behavior in the case of the emulsions, as expected.

In the case of emulsions, the parameters $R_{r}$ and $E_{r}$ exhibited variations over time. The greatest variations occurred in the lower concentration emulsions. In the case of the 10% concentration, the $R_{r}$ value almost doubled, and then decreased steadily until the end of the test. The other concentrations (20%, 27%, and 50%) remained practically constant. In the case of $E_{r}$ and the concentration of 10%, the value also increased to double its value, but remained constant for the rest of the test. The concentrations of 20% and 27% also varied, but to a lesser extent. This behavior may be a consequence of changes in the emulsion, mainly the coalescence of water droplets. The results also show that the relative energy appeared to be more sensitive to changes in the properties of the emulsion.

In turn, the propagation velocity ($c_{12}$), as shown in Figure 3c, is a fairly stable property. The measured speeds were almost constant throughout the test time and well differentiated according to the concentration. Again, the 10% concentration had the greatest variation, showing an increase in speed toward the end of the test. However, the increase was very small, less than 0.3% compared to the value at the beginning of the test. This increase may be a consequence of a higher concentration of water droplets in the lower part of the sample where the measurement device is located.

**Figure 4:**
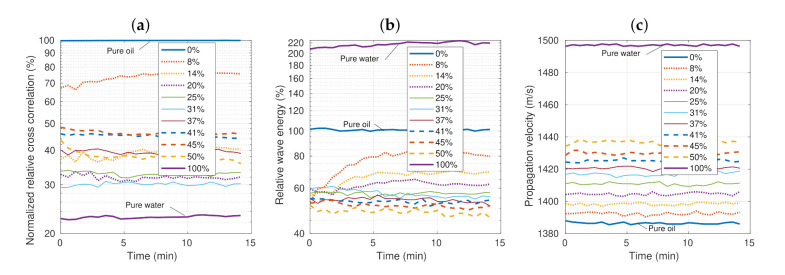
Experimental results of the short-term test: the relative cross-correlation (**a**), relative wave energy (**b**), and propagation velocity (**c**) as a function of time at $22\pm 0.2{\;}^{\circ }$C.

Figure 4 shows the measured acoustic parameters for the short-term test. In addition to pure water and oil, nine emulsions with a water concentration between 8% and 50% were tested. The test time was approximately 14 min. The results of $R_{r}$ ( Figure 4a) and $E_{r}$ (Figure 4b) showed an unexpected behavior, with variations over time that were greater at lower concentrations. The curves of the different concentrations were mixed, making them difficult to differentiate. This behavior is consistent with the start of the long-term tests, showing that the measuring device had good precision. In the case of $c_{12}$, constant values can be seen, with very small temporal variations, and the different concentrations were perfectly differentiable. These results are promising due to the stability between the propagation velocity and water concentration. They anticipate a good sensor resolution.

**Figure 5:**
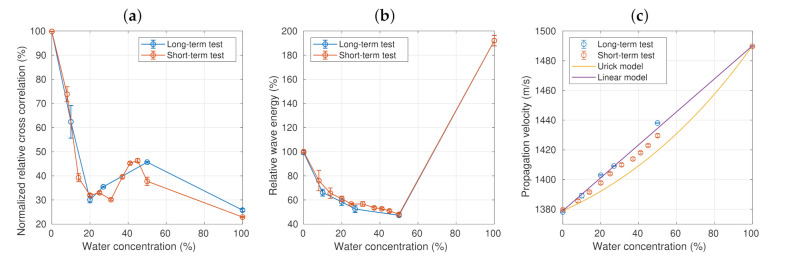
The experimental results as a function of the water concentration: (**a**) relative cross correlation, (**b**) relative wave energy, and (**c**) propagation velocity at $22\pm 0.2{\;}^{\circ }$C.

The calculated acoustic parameters for both long- and short-term tests (Figure 3 and Figure 4, respectively) are presented in the form of the mean and standard deviation of all temporal samples, as shown in Figure 5. Figure 5a shows the relative cross-correlation ($R_{r}$) as a function of the water concentration (in percentage) for both the long-term and short-term tests. For small concentrations, the $R_{r}$ value decreased from 1 to a minimum value of about 0.3 at a concentration of 20%. This behavior was the same in both tests. In the range between 20% and 50%, $R_{r}$ increased slightly until it reached a maximum value of 0.46 just before 50%. The values obtained with pure water for the two tests were close to 0.26. In this range, the short-term test exhibited a wider variation in $R_{r}$ compared to the long-term test.

The monotonic decreasing behavior observed at low concentrations, in both tests, provides a useful measuring range between 0 and 20%. These results suggest good sensitivity and temporal stability. Taking into account that most emulsions found in the oil industry have a volumetric water concentration of less than 20%, this result is relevant. However, additional studies are needed to support the results yielded here.

Figure 5b shows the relative wave energy as a function of the concentration for both the long- and short-term tests. In this case, the relative energy value continuously decreased in the entire range, from 1.0 for pure oil to 0.45 for an emulsion with water concentration of 50%. The behavior was the same in both tests; however, there were small numerical differences, which were a consequence of the emulsion physical properties varying in time.

Figure 5c shows the propagation velocity as a function of the concentration for both the long- and short-term tests. The calculation methodology was explained in Section 2.2. The propagation velocity increased from the minimum value measured with pure oil to the maximum value for water. This variation was linear with respect to the water concentration, suggesting that it can be modeled by a simple mixing rule, albeit different from Urick’s model [36]. The behavior was the same in both tests, with small differences in the numerical values. Furthermore, the results suggest good sensitivity, since the speed changes detected were as small as 3.88 m/s, corresponding to 0.28% of the propagation velocity in pure oil.

The estimation of the water concentration from the measured parameters requires an error analysis. In the case of relative cross-correlation (Figure 5a), the usable measurement range was 0–20% of the water by volume. However, there were few measurements in this range to fit a model. In the case of energy (Figure 5b), this is a very stable property; however, the usable measurement range is similar to that of correlation (0–20%) because the slope is low above 20% of water, which leads to a low sensitivity. The fitting of a third order polynomial allows determining the water concentration with a relative error of less than 18% in the range 0–20%. Above 20% water content, the error increased considerably, becoming even greater than 50%.

The most promising parameter is the propagation velocity, because the behavior was almost linear over a wider concentration range (0–50%). This behavior allows proposing a simple model to represent the data. The linear model proposed consists of measuring the propagation velocity for the pure substances (pure oil and pure water) and tracing a straight line connecting the extremes to describe the relationship between the propagation velocities and the different contents of water in water-in-oil emulsions.

This linear model is represented in Figure 5c. During the experiments, the propagation velocity was measured in different emulsions in which the water content was known. Thus, the measured propagation velocities were used to estimate the water concentrations, using the linear model. Table 1 shows the concentration values obtained using the proposed model, compared to the known concentrations (amount of water added). Errors of less than 9% and 26% for the long- and short-term tests, respectively, were obtained for the full range of measurement. In the case of the short-term test, the values were always lower than expected.

## 4. Conclusions

A multiple-scattering ultrasonic sensor was developed and applied to monitor water-in-crude-oil emulsions with volume water concentrations of up to 50%. Backscattering signals were analyzed using the cross-correlation and wave energy parameters, both exhibiting dependence on the water concentration in the emulsion. Tests carried out for long (longer than 3 h) and short (15 min) periods of time showed similar behavior, with a difference of less than 15% in the numerical values and stability.

The relative cross-correlation showed a steadily decreasing behavior, with a steep slope, at low concentrations. This behavior allows for determination of the amount of water in the emulsion with good resolution in the concentration range between 0% and 20%. Conversely, the relative energy of the wave decreased continuously over the analyzed concentration range. This allows characterizing the emulsion in the concentration range between 0% and 50%, although with a lower resolution compared with in the case of correlation. The energy of the backscattered signal was also shown to be a time-stable property that was greater than that of the correlation.

The sensor allowed measuring the propagation velocity, which is a property directly related to the concentration in emulsions with low scattering. In this case, a propagation velocity proportional to the water concentration was observed. This linear behavior, which did not agree with Urick’s mixing model, is promising for water content monitoring. Percentage relative errors less than 9% and 26% for the long- and short-term tests, respectively, were obtained.

The sensor is robust and easy to manufacture. It operates in the frequency range of the transducer. Other working frequencies can be used, as long as the transducer is changed. The rows of scatterers generate echoes in specific positions that allow determination of the propagation velocity, but avoid most of the disadvantages associated with a design based on delay lines. The signal processing is simple, with algorithms that allow for implementation in a microcontroller or some other embedded system of low computational power.

The results show that the sensor is promising for online and real-time monitoring of the water content in water-in-crude-oil emulsions. The technique requires the mean size of the water droplets in the emulsion (dispersed phase) to be less than the wavelength (likely an order of magnitude smaller). However, additional research should be carried out to evaluate the best configuration of the scatterers, the working frequency, and the possibility of measuring emulsions in motion (flowing through the sensor).

Finally, compared to the traditional technologies (emission–reception and pulse–echo), the sensor presented here was more sensitive and performed better with emulsions. In the case of emulsions with a relatively large droplet size, where the signal is highly attenuated such that the receiver cannot detect it, the multiple dispersion sensor is more likely to continue measuring. This subject will be studied in a future work.

## Figures and Tables

**Table 1:** The known (added water) and estimated (calculated using the linear model and the measured propagation velocity) water concentrations and the percentage relative error.

Long-Term Test									
Known water concentration (%)	10	20	27	50					
Estimated water concentration (%)	9.12	21.56	27.15	53.22					
Error (%)	−8.8	7.8	0.5	6.4					
**Short-Term Test**									
Known water concentration (%)	8	14	20	25	31	37	41	45	50
Estimated water concentration (%)	5.95	11.32	16.88	22.50	27.90	31.40	35.19	39.57	45.59
Error (%)	−25.7	−19.2	−15.6	−10.0	−10.0	−15.1	−14.2	−12.1	−8.8
